# The regulatory loop of COMP1 and HNF-4-miR-150-p27 in various signaling pathways

**DOI:** 10.3892/ol.2014.2643

**Published:** 2014-10-27

**Authors:** WEIWEI NIE, JUN GU, ZEXING WANG, DONGHAI LI, XIAOXIANG GUAN

**Affiliations:** 1 Department of Medical Oncology, Jinling Hospital, Southern Medical University, Guangzhou, Guangdong 510282, P.R. China; 2 Department of Extramammary, Jinling Hospital, Medical School of Nanjing University, Nanjing, Jiangsu 210002, P.R. China; 3 Department of Medical Oncology, Jinling Hospital, Medical School of Nanjing University, Nanjing, Jiangsu 210002, P.R. China; 4 Jiangsu Engineering Research Center for MicroRNA Biology and Biotechnology, State Key Laboratory of Pharmaceutical Biotechnology, School of Life Sciences, Nanjing University, Nanjing, Jiangsu 210093, P.R. China

**Keywords:** bioinformatics analysis, miR-150, p27, signaling pathway

## Abstract

MicroRNAs (miRNAs) are short regulatory RNAs that negatively modulate protein expression at the post-transcriptional level. Additionally, they have been associated with the pathogenesis of a number of types of cancer. In the current study, two target sites for miR-150 were determined within the 3′-untranslated region of p27^Kip1^ (hereafter referred to as p27) mRNA, and it was determined that ectopic overexpression of miR-150 led directly to p27 downregulation in cancer cells. These findings indicate that miR-150 may be a novel regulator of p27 expression. In the databases of the University of California, Santa Cruz (UCSC) and Match online, two common transcription factors were identified for miR-150 and p27: Cooperates with myogenic proteins 1 (COMP1) and hepatocyte nuclear factor-4 (HNF-4). Using the Database for Annotation, Visualization, and Integrated Discovery (DAVID), it was determined that p27 is involved in pathways regulated by the target genes of miR-150. Therefore, these results suggest that there may be a regulatory loop between COMP1 and HNF-4-miR-150-p27. Additional functional studies are required to understand the molecular basis for the formation of this circuit loop, and provide an insight into the development of innovative therapies targeting specific tumor markers.

## Introduction

MicroRNAs (miRNAs) are non-coding (nc) RNAs of ~22 nucleotides in length, the mutation or deregulated expression of which are associated with a number of types of human cancers. Furthermore, miRNAs act as tumor suppressor genes or oncogenes (1). Profiling experiments have determined that changes in the levels of miRNA expression are more effective predictors of tumor type than changes in mRNA expression levels. This led to the identification of miRNA signatures for specific types of cancer (2). Depending on their target genes, miRNAs may serve to stimulate or suppress tumor formation and growth.

p27 is an atypical tumor suppressor that regulates the G_0_/S phase transition by binding to and regulating the activity of cyclin-dependent kinases (CDKs) (3). As a member of the Cip/Kip family, p27 binds to the catalytic cleft of the cyclin/CDK complex to prevent ATP recognition. Notably, in the majority of human cancers the expression levels of p27 protein are reduced or the protein is mislocalized, which are associated with a poor prognosis.

Additionally, translation of p27 is downregulated by miRNAs via their interactions with the 3′-untranslated region (UTR) of mRNAs (4). It has been reported that miR-221 and miR-222 bind to the 3′-UTR of p27 mRNA and inhibit their translation. Hence, the miRNA-mediated inhibition of p27 translation may be a novel mechanism that reduces the expression levels of p27 in certain types of human cancer. miR-150 is one of the most extensively investigated miRNAs. It acts as a tumor suppressor, and the downregulation of miR-150 induces the activation of the PI3K-AKT pathway, leading to the activation of telomerase and the immortalization of cancer cells (5). In the current study, the association between miR-150 and p27 was investigated in order to elucidate the regulatory mechanism of p27 at transcriptional and post-transcriptional levels.

## Materials and methods

### Bioinformatics analysis

Analysis of the predicted miRNA targets was undertaken using algorithms from TargetScan (http://genes.mit.edu/targetscan/). The biological characters of these target genes were analyzed using the Database for Annotation, Visualization and Integrated Discovery (DAVID; http://david.abcc.ncifcrov/). In the University of California, Santa Cruz database (UCSC; http://genome.ucsc.edu), a sequence between -2000 and 2000 bp of miR-150 was selected. Using the Match database, the transcription factors (TF1) that may bind to the upstream sequence of miR-150 were identified. In the same manner, the transcription factors (TF2) that regulate p27 expression were identified. By comparing TF1 and TF2, common transcription factors were identified between miR-150 and p27.

### Cell culture

THP1 and MCF-7 cell lines (Shanghai Institutes for Biological Sciences, Chinese Academy of Sciences, Shanghai, China) were cultured in RPMI-1640 medium (Invitrogen Life Technologies, Carlsbad, CA, USA) supplemented with 10% fetal bovine serum (Gibco-BRL, Carlsbad, CA, USA) at 37°C in a humidified atmosphere with 5% CO_2_.

### Plasmid construction and luciferase assay

The entire human p27 3′-UTR was amplified by polymerase chain reaction (PCR) using human genomic DNA as a template. The PCR products were inserted into the p-GL3-report plasmid (The University of Tokyo, Tokyo, Japan). Correct insertion was confirmed using sequencing. For the luciferase reporter assays, the cells were cultured in six-well plates. Each culture was transfected with 2 μg firefly luciferase reporter plasmid (Jiangsu Diabetes Center, State Key Laboratory of Pharmaceutical Biotechnology, Nanjing University, Nanjing, China), 2 μg β-galactosidase expression vector (Ambion Life Technologies, Carlsbad, CA, USA), and equal amounts of scrambled ncRNA, a synthetic RNA oligonucleotide mimicking miR-150 precursors (pre-miR-150), or a chemically modified antisense oligonucleotide designed to specifically target mature miR-150 (anti-miR-150) using Lipofectamine 2000 (Invitrogen Life Technologies). The β-galactosidase vector was used as a transfection control. At 24 h post-transfection, the cells were assayed using a luciferase assay kit (Promega, Madison, WI, USA). Data are representative of three independent experiments performed on different days.

### Overexpression or knockdown of miR-150

miR-150 overexpression was achieved by transfecting cells with pre-miR-150, whereas miR-150 knockdown was performed by transfecting cells with anti-miR-150. An equal amount (200 pmol) of scrambled ncRNA served as the negative control. MCF-7 cells were seeded in six-well plates or 60-mm dishes and then transfected the following day using Lipofectamine 2000, according to the manufacturer’s instructions.

### RNA isolation and reverse transcription quantitative PCR (RT-qPCR)

Total RNA was extracted from the cultured cells using TRIzol (Invitrogen Life Technologies) according to the manufacturer’s instructions. For RT-qPCR analysis of p27 and β-actin, cDNA was reverse transcribed from1 μg total RNA using oligdT and Thermoscript (Takara, Dalian, China). qPCR analyses of p27 and β-actin were performed on an ABI 7300 Sequence Detection System (Applied Biosystems, Foster City, CA, USA) using SYBR green dye (Invitrogen Life Technologies). The reaction included 1 μl cDNA, 1× QuantiTect SYBR Green PCR master mix (Invitrogen Life Technologies), and 0.5 μM of each sense and antisense primer, with a final volume of 20 μl. All PCR was run in triplicate. Threshold cycles (CT) were determined using fixed threshold settings. Primer sequences were as follows: Forward, 5′-AGAGCCAACAGAACAGAAGAA-3′, and reverse, 5′-AGAGGCAGATCATTTAAGAGTG-3′ for p27; and forward, 5′-AGGGAAATCGTGCGTGAC-3′ and reverse, 5′-CGCTCATTGCCGATAGTG-3′ for β-actin.

Assays to quantify the levels of mature miR-150 were performed using TaqMan microRNA probes (Applied Biosystems) as previously described (6). Briefly, 5 μl total RNA was reverse transcribed to cDNA using AMV reverse transcriptase (Takara) and a stem-loop RT primer (Applied Biosystems). qPCR was performed using a TaqMan PCR kit (Takara) on the ABI 7300 Sequence Detection System. All PCR experiments, including the no template controls, were run in triplicate. The miRNA expression levels were normalized using U6 snRNA as an internal control (7). The relative levels of miR-150 to U6 were calculated using the 2^−ΔΔCT^ equation, in which ΔCT = CT_miR-150_ - CT_U6_.

### Western blotting

The expression levels of p27 protein were quantified via western blot analysis of the whole cell extracts using a monoclonal rabbit anti-human antibody against p27 (1:1,000; 3688, Cell Signaling Technology, Inc., Danvers, MA, USA). Samples were normalized by blotting with a polyclonal rabbit anti-human antibody against α-tubulin (1:1,000; 2144, Cell Signaling Technology, Inc.).

### Statistical analysis

All images of western blotting and semi-quantitative RT-PCR are representative of at least three independent experiments. RT-qPCR and luciferase reporter assays were performed in triplicate. Data are presented as the mean ± standard deviation of at least three independent experiments.

## Results

### Bioinformatics analysis of miR-150

Using TargetScan, 275 target genes were identified for miR-150 (Table I), including p27. By the same method, it was determined that a number of miRNAs, including miR-150, may bind to the 3′-UTR of p27 mRNA.

### miR-150 is potent suppressor of p27 expression

**Figure 1:**
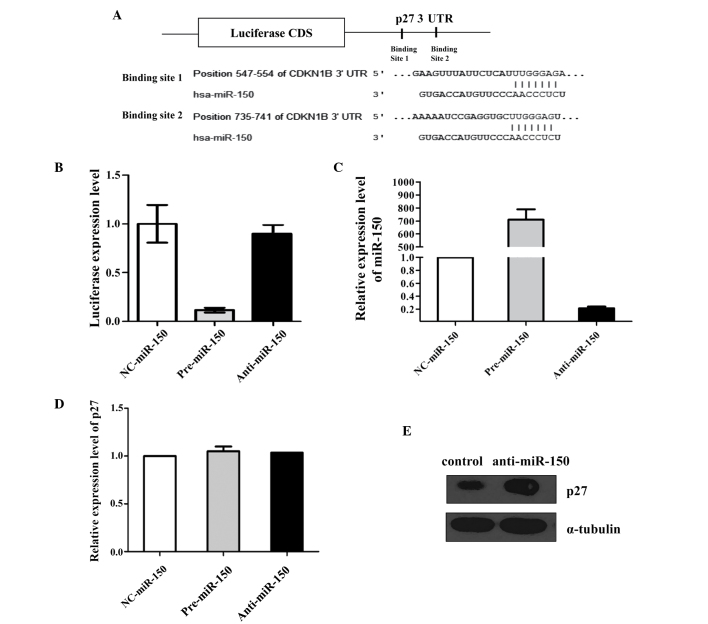
Identification of conserved microRNA (miR)-150 binding sites within the p27 3′-UTR, and regulation of p27 expression by miR-150 at the mRNA and protein levels. (A) Diagram of the luciferase reporter plasmid carrying the firefly luciferase coding sequence fused to the human p27 3′-untranslated region (UTR) with wild-type miR-150 binding sites. (B) Direct recognition of the p27 3′-UTR by miR-150. Firefly luciferase reporters containing the p27 3′-UTR were cotransfected into MCF-7 cells with scrambled ncRNA, pre-miR-150 or anti-miR-150. At 24 h post-transfection, the cells were assayed using a luciferase assay kit. The y-axis shows arbitrary units representing relative luciferase activity. (C and D) Overexpression or knockdown of miR-150. MCF-7 cells were seeded in six-well plates and transfected the following day with scrambled ncRNA, pre-miR-150 or anti-miR-150 (200 pmol each) using Lipofectamine 2000. miR-150 levels were evaluated by reverse transcription quantitative polymerase chain reaction at 24 h post-transfection. For comparison, the expression levels of miR-150 in ncRNA-transfected cells were arbitrarily set as 1. The y-axis shows arbitrary units representing relative miR-150 expression levels. (E) Western blot analysis of the p27 protein levels in THP1 cells that were untreated or treated with anti-miR-150 at 48 h post-transfection. The results are presented as the mean ± SD of three independent experiments.

miRNA associates with mRNA via complementary Watson-Crick base pairing. The common criteria to determine whether a transcript is a target for a miRNA is base pairing between the ‘seed’ and target, and an inverse correlation between the miRNA expression levels and its target levels. The seed sequence (the binding sequence of the target mRNA) has varying degrees of complementarity with the miRNA (8). The results of the present study revealed that the p27 3′-UTR harbors two sites that are likely recognized by miR-150, located at nucleotides 547–554 and 735–741 of the p27 mRNA (NM_004064.0) (Fig. 1A).

### p27 is a direct target of miR-150

To investigate whether the negative regulation of p27 expression by miR-150 occurred via binding to the aforementioned complementary sites within the 3′-UTR of the p27 mRNA, the whole of the p27 3′-UTR was added to the downstream position in a firefly luciferase reporter plasmid. Subsequently, the plasmid was transfected into MCF-7 cells along with a transfection control plasmid (β-gal) and pre-miR-150, anti-miR-150 or scrambled ncRNA. The overexpression of miR-150 resulted in a significant decrease in the level of luciferase reporter activity (normalized to β-gal activity) compared with that of the cells transfected with the scrambled ncRNA. However, the inhibition of miR-150 resulted in a less significant increase in reporter activity (Fig. 1B). This finding suggests that these binding sites strongly contribute to the miRNA-mRNA interaction that mediates the post-transcriptional inhibition of p27 expression.

This result was validated by performing an RT-qPCR assay. MCF-7 cells were transfected with ncRNA, pre-miR-150 or anti-miR-150 and analyzed for expression of miR-150 and p27 mRNA using RT-qPCR at 24 h post-transfection (Fig. 1C and D). All of the cells that were transfected with pre-miR-150 demonstrated increased levels of miR-150 relative to those in the cells transfected with ncRNA. By contrast, transfection with anti-miR-150 resulted in a significant reduction in the expression levels of miR-150 in MCF-7 cells. Western blot analysis of p27 protein levels in THP1 cells demonstrated that the expression of p27 protein was elevated following transfection with anti-miR-150 (Fig. 1E).

### Analysis of transcription factors for miR-150 and p27 expression

**Figure 2:**
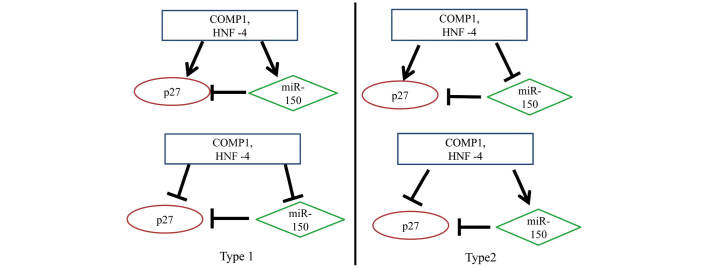
Simplified representation of Type I and Type II circuits. The master transcription factors are cooperates with myogenic proteins 1 (COMP1) and hepatocyte nuclear factor-4 (HNF-4), and p27 is the target gene. Inside each circuit, → indicates transcription activation and ⊥ indicates post-transcriptional repression.

The transcription factors for miR-150 and p27 expression included Oct-1, AP-1, NF-1, Pax-4, USF, cooperates with myogenic proteins 1 (COMP1), hepatocyte nuclear factor (HNF)-1, SOX-9, Pax-6, Pax-4, HNF-4, Cart-1, Elk-1, HNF-3β and FOXD3. Using the UCSC and Match databases, COMP1 and HNF-4 were identified as common transcription factors for miR-150 and p27 expression. Using DAVID, it was determined that p27 was involved in pathways regulated by miR-150. Therefore, these results have revealed a regulatory loop involving COMP1 and HNF-4-miR-150-p27. The transcription factors COMP1 and HNF-4 may enhance or attenuate the regulation of miR-150 and p27. Due to the computational procedure adopted to identify the regulatory loop based on sequence analysis only, it was not possible to determine whether the actions of COMP1 and HNF-4 were excitatory or inhibitory. Therefore, we propose there are two types of interactions (Fig. 2) (9). The results of the current study attained that there are two kinds of microRNA-transcription factor feed-forward regulatory circuits, which are referred to as Type I and Type II circuits (10). Tsang *et al* (10) reported that Type I circuits stabilize the steady state production of a protein by dumping transcriptional fluctuations, whereas Type II (coherent) circuits lead to the reinforcement of transcriptional regulation at the post-transcriptional level. COMP1 and HNF-4 act as master transcription factors, inducing the expression of miR-150 and the joint target p27, which in turn, is repressed by miR-150.

### Bioinformatics analysis of the characters of miR-150

**Figure 3:**
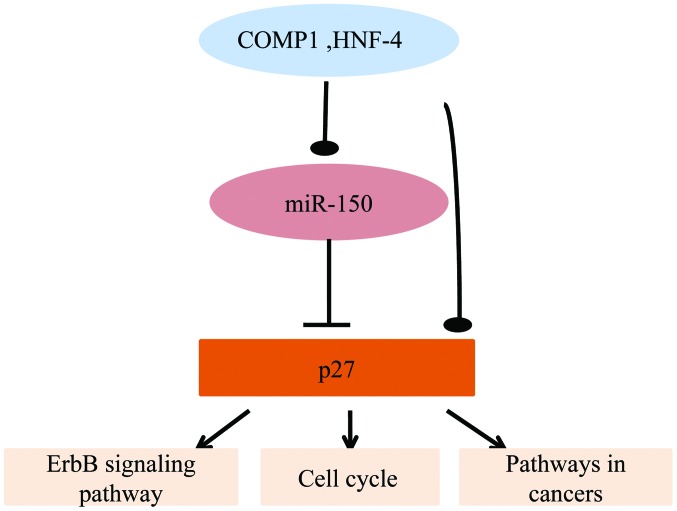
Graphical representation of the network consisting of cooperates with myogenic proteins 1 (COMP1) and hepatocyte nuclear factor-4 (HNF-4)-microRNA (miR)-150-p27, including certain important signaling pathways. Arrows indicate activation. Lines with blunt ends indicate post-transcriptional repression. Lines with an oval on the end indicate the uncertain transcription regulation of COMP1 and HNF-4 to miR-150 or p27, which may be active or supressive.

The target genes of miR-150 were imported into DAVID. The gene ontology (GO) biological processes (BP), GO molecular functions (GOMF), and Kyoto Encyclopedia of Genes and Genomes (KEGG) pathways were analyzed. It was determined that miR-150-p27 participated in important GOBP, including the regulation of microtubule cytoskeleton organization, the regulation of phosphorus metabolic processes, the negative regulation of transcription and programmed cell death. Additionally, miR-150-p27 was involved in important GOMF, including protein kinase regulator activity and kinase regulator activity. In addition, they were involved in certain important signaling pathways, including the ErbB signaling pathway and pathways involved in cancer and the cell cycle (Table II). Hence, we propose that the regulatory loop of COMP1 and HNF-4-miR-150-p27 has a complicated role in these critical biological processes (Fig. 3).

## Discussion

Using the UCSC and Match databases, two common transcription factors were identified for miR-150 and p27: COMP1 and HNF-4. In addition, the possible biological roles of this regulatory loop were analyzed using DAVID. Furthermore, it was determined that miR-150-p27 participated in the regulation of microtubule cytoskeleton organization, the regulation of phosphorus metabolic processes, and the negative regulation of transcription and programmed cell death. miR-150-p27 was also involved in the regulation of protein kinase regulator activity and kinase regulator activity. Notably, miR-150-p27 participated in crucial signaling pathways, including the ErbB signaling pathway and pathways involved in cancer and the cell cycle. Therefore, the results of this study indicate that there may be a regulatory loop between COMP1 and HNF-4-miR-50-p27. Further study is required to test this hypothesis and comprehensively examine the regulation of p27 expression by miR-150.

Expression of p27 is regulated at multiple levels, including transcription, mRNA stability, translation, proteolysis, and subcellular localization. Several mechanisms of regulation may coexist in a single cell depending on the cell type, extracellular stimuli and biological circumstances (11). miRNAs have been recognized as key regulators of p27 mRNA expression. The first indications that miRNAs play a role in the expression of p27 were observed in *Drosophila* (12).

The results of the current study, which identified p27 as a target of miR-150 in carcinoma cell lines, are in agreement with the dynamic view of the miRNA-mediated regulation of gene expression. The association between miRNAs and target mRNAs is not a ‘one to one’ association, as the same mRNA can be regulated by more than one miRNA. Furthermore, the degree to which miRNAs target a specific 3′-UTR are strongly determined by the specific cellular environment (13).

miR-150 is specifically expressed in mature lymphocytes. A major predicted target of miR-150 is c-Myb, a transcription factor that controls multiple steps of lymphocyte development (13). Furthermore, control of the Notch pathway through miR-150 may have an important effect on T-cell development. The results of the current study suggest that miR-150 may regulate p27 at the post-transcription level.

Investigation of transcription factor binding sites indicates that transcriptional and post-transcriptional regulatory interactions can be predicted *in silico* by searching for over-represented short sequences of nucleotides present in promoters or 3′-UTRs (14). Therefore, the aim of the current study was to use computational tools to generate a list of regulatory loops in wfhich a master transcription factor regulated a miRNA together with target genes (15). As a result, a regulatory loop involving COMP1 and HNF-4-miR-150-p27 was revealed. The primary purpose of this study was to systematically investigate the associations between the transcriptional and post-transcriptional network interactions of p27. A regulatory loop has been previously validated for MYC-E2F2/E2F1-miR-20a (13), which is a Type I.

In conclusion, additional functional studies are required to understand the molecular basis of the formation of this regulatory loop, and to provide insight towards the development of innovative therapies targeting specific tumor markers.

## Figures and Tables

**Table I:** Target genes of miR-150 in the TargetScan database.

miRNA	Target genes
miR-150	MYB, ADIPOR2, IRAK2, PDCD4, C13orf34, ENSA, C22orf46, CBL, SV2B, CALCR, GABRA4, MTCH2, ITGB3, DNAJB7, C7orf68, SP5, TADA1, ATP6V1H, USP13, ZNF229, ZEB1, EPB41L5, WTAP, GLE1, LTBP2, MBD6, BASP1, ZBTB4, OSBPL9, ADAM19, RORB, ENTPD1, ELOVL3, FOXD3, CAST MDGA1, TRIM66, **CDKN1B**, CPD, CMTM6, NKX24, FADS1, PLP2, PISD, EIF4B, C7orf64, GCM2, TP53, PIK3AP1, TMEM48, SORCS3, KIAA1274, VSIG10, PAN2, CAMK2G, MMP14, GABRG2, GGNBP2, CNPPD1, UST, MBTD1, CACNA1G, LRRC58, PPFIA3, TBC1D14, PAPPA, PRICKLE2, EP300, GRIPAP1, FTO, ARIH2, MRPL27, FAM134C, S TX5, UBFD1, EBF3, IRF2BP2, JPH2, C1orf183, EGR2, RIIAD1, SHISA4, ZCCHC17, ZMAT2, ETF1, ITPRIPL2, RAB11A, SH3BP5L

Bold text indicates the the offical full name of p27.

**Table II:** KEGG pathways regulated by miR-150 target genes.

KEGG Pathway	Involved gene
**hsa04012: ErbB signaling pathway**	PRKCA, CDKN1B, CAMK2G, GSK3B, CBL, GAB1, MAP2K4, ELK1, AKT3
hsa05213: Endometrial cancer	GSK3B, TP53, ELK1, AKT3, APC
hsa05217: Basal cell carcinoma	GSK3B, TP53, FZD4, APC
hsa05210: Colorectal cancer	ACVR1B, GSK3B, TP53, FZD4, AKT3, APC
**hsa05220: Chronic myeloid leukemia**	ACVR1B, CDKN1B, CBL, TP53, AKT3
hsa04310: Wnt signaling pathway	PRKCA, EP300, CAMK2G, GSK3B, BTRC, PRICKLE2, TP53, FBXW11, FZD4, APC
hsa04912: GnRH signaling pathway	PRKCA, MAPK13, CAMK2G, MAP2K4, ELK1, MMP14
hsa04722: Neurotrophin signaling pathway	IRAK2, MAPK13, CAMK2G, GSK3B, GAB1, TP53, AKT3
**hsa05215: Prostate cancer**	CDKN1B, EP300, GSK3B, TP53, AKT3
hsa04340: Hedgehog signaling pathway	GSK3B, BTRC, FBXW11
hsa04664: Fc epsilon RI signaling pathway	PRKCA, MAPK13, MAP2K4, AKT3
**hsa05222: Small cell lung cancer**	COL4A4, CDKN1B, TP53, AKT3
hsa04514: Cell adhesion molecules (CAMs)	GLG1, PECAM1, PVRL2, NFASC, NLGN3, NEGR1
hsa05212: Pancreatic cancer	ACVR1B, TP53, AKT3
hsa04010: MAPK signaling pathway	PRKCA, ACVR1B, DUSP3, MAPK13, MAP2K4, CACNA1G, TP53, ELK1, AKT3, MAP3K12
**hsa05200: Pathways in cancer**	PRKCA, COL4A4, ACVR1B, CDKN1B, EP300, GSK3B, CBL, SLC2A1, TP53, FZD4, AKT3, APC
**hsa04110: Cell cycle**	CDKN1B, EP300, GSK3B, TP53
hsa04120: Ubiquitin mediated proteolysis	BTRC, CBL, FBXW11, UBE2R2
hsa04060: Cytokine-cytokine receptor interaction	CSF3, ZFP91, ACVR1B, EDA, CCL5

Bold text indicates pathways that may be regulated by p27. KEGG, Kyoto Encyclopedia of Genes and Genomes.
